# Sex-Specific Association of Serum Anti-Oxidative Capacity and Leukocyte Telomere Length

**DOI:** 10.3390/antiox10121908

**Published:** 2021-11-28

**Authors:** Eva Hassler, Gunter Almer, Gernot Reishofer, Gunther Marsche, Harald Mangge, Hannes Deutschmann, Markus Herrmann, Stefan Leber, Felix Gunzer, Wilfried Renner

**Affiliations:** 1 Division of Neuroradiology, Vascular and Interventional Radiology, Department of Radiology, Medical University Graz, 8036 Graz, Austria; eva.hassler@medunigraz.at (E.H.); hannes.deutschmann@medunigraz.at (H.D.); stefan.leber@medunigraz.at (S.L.); felix.gunzer@medunigraz.at (F.G.); 2 Clinical Institute of Medical and Chemical Laboratory Diagnostics, Medical University Graz, 8036 Graz, Austria; gunter.almer@medunigraz.at (G.A.); harald.mangge@medunigraz.at (H.M.); markus.herrmann@medunigraz.at (M.H.); wilfried.renner@medunigraz.at (W.R.); 3 Department of Radiology, Medical University Graz, 8036 Graz, Austria; 4 BioTechMed Graz, 8010 Graz, Austria; 5 Division of Pharmacology, Otto Loewi Research Center, Medical University of Graz, 8036 Graz, Austria; gunther.marsche@medunigraz.at

**Keywords:** leucocyte telomere length, telomere shortening, oxidative stress, telomerase, total antioxidative capacity, lifestyle changes

## Abstract

Telomeres are a crucial factor in the preservation of genomic integrity, and an elevated risk for diseases such as cancer and cardiovascular events is related to shortened telomeres. However, telomere deterioration could be caused by factors such as chronic oxidative stress and inflammation, which are promoted by an imbalance among reactive oxygen species (ROS) and antioxidants. In this cross-sectional study, we investigated the relationship between telomeres and oxidative stress. The serum leucocyte telomer length (LTL), serum total antioxidant capacity (TAC) and the total serum lipid panel of 180 healthy athletic volunteers (90 males, 90 females) were measured Additionally, a questionnaire about sports behaviour and the type of training was completed. We observed a positive significant relation between serum LTL and TAC in the male group (cc = 3.4/*p* = 0.001) but not in females. There was no statistically significant correlation between age and physical activity and LTL in both groups. This is the first cross sectional study demonstrating an association between total serum TAC and LTL in healthy males, but interestingly, not in the females. Nevertheless, these results should be interpreted as preliminary, and further studies in independent cohorts are needed to investigate the sex-specific effects of oxidative stress on telomere length and telomerase activity.

## 1. Introduction

Telomeres consist of tandem repeats of the hexanucleotide TTAGGG sequence and an associated complex consisting of six proteins for their own protection. They are positioned at the edges of chromosomal DNA [1] and as part of the heterochromatin they are essential for chromosomal stability and cellular integrity [2,3,4].

Over time telomeres shorten by 50 to 100 nucleotides with each cell division [5]. A critical telomere length leads to chromosomal instability, cell senescence and apoptosis in normal as well as pre-malignant cells. These factors also promote age-related degenerative diseases and cancer. Moreover, leukocyte telomere length (LTL) has been suggested as a biomarker of biological age, and shorter telomere length (TL) is linked to a higher risk for several diseases, such as cancer and cardiovascular diseases [4].

Oxidative stress and inflammation seem to be possible contributors to telomere deterioration [6,7,8]. It is an imbalance among reactive oxygen species (ROS) and antioxidants followed by an excessive ROS production. Oxidative stress not only causes damage to DNA but also to lipids and proteins, and it is involved in the pathogenesis of atherosclerosis [9]. Houben et al. showed an increased rate of telomere shortening under conditions of oxidative stress [10]. Telomeres are highly sensitive to hydroxyl radicals, leading to DNA breakage, because greater telomere stretches are clipped which each replication, especially in haematopoietic stem cells. This represents an underlying mechanism of telomere shortening [11].

An important enzyme with the main function of elongation and maintaining telomeres is the reversed transcriptase telomerase. It is composed of two main parts, first a telomerase RNA component (TERC), which contains the template for the synthesis of telomeres, and the additional catalytic protein the human telomerase reverse transcriptase (hTERT). When the telomerase binds to the telomeres, TERC is the matrix for TERT, which then can replicate the telomere sequences [12].

Processes promoting healthy ageing are influenced by changes in telomerase activity in different cell types, such as lymphocytes. Additionally, negative lifestyle factors and an elevated level of serum LDL seem to promote cardiovascular disease and affect telomerase function [13]. Earlier longitudinal studies have shown that improvements in nutrition and lifestyle are associated with enhanced telomerase activity [13]. In another pilot study it was shown that life-style interventions that are known to reduce the risk of chronic diseases or aging effects such as regular exercise can influence telomere shortening positively. However, the regulative mechanisms are mostly unknown [14].

Furthermore, chronic oxidative stress is linked to cardiovascular and chronic inflammatory diseases such as inflammatory bowel disease and diabetes type 2, and it is known that in subjects with such chronic diseases, telomeres shorten faster [10,15,16]. Decreased serum total antioxidant capacity (TAC), reduced LTL and increased plasma 8-OHdG concentrations have been found in patients with Alzheimer’s disease in comparison to an age-matched group of non-demented subjects [17]. Garcia et al. showed a relation between the dietary total antioxidant capacity and the LTL in a child and adolescent population including both sexes [18]. Concordant, an in vitro study, showed that reactive oxygen species (ROS) decreased the level of nuclear hTERT and telomerase activity in endothelial cells, leading to senescent phenotype development [19]. The antioxidant defence system helps to counteract reactive oxygen (ROS) and nitrogen species (RNS) in living organisms. It consists of a combination of different endogenous antioxidant enzymes, proteins such as albumin, high-density lipoproteins, glutathione (GSH) and more, and incorporates dietary substances such as vitamins C and E or carotenoids. These substances work collectively to inactivate oxidative agents for the prevention of oxidative damage to macromolecules [20].

Several methods have been established for the measurement of TAC. The majority of these methods are based on the capacity to reduce oxidised compounds. The combination of the activity of all antioxidants in an organic sample into a single value represents the main strength of TAC measurement. Instead of a sum of measurable substances it provides one integrated parameter [21].

Moreover, prior studies indicated that LTL is a dynamic factor that could be modified by lifestyle changes, such as nutrition or physical activity [18]. The Mediterranean diet pattern especially seems to be positively related to TL [6,10]. Physical activity also seems to have a positive influence on TL and TAC. Recent studies showed that master athletes have longer telomeres and higher TAC than non-athletes. Additionally, the training intensity can play an important role [22,23].

Hence, the aim of our study was to investigate the relationship between leukocyte telomere length and oxidative stress in healthy and physically active, non-professional athletes. We also focused on a possible influence on telomere length and serum TAC variations due to differences in the extent and type of physical activity, and possible differences in both groups of sex.

## 2. Materials and Methods

### 2.1. Subjects

The study was approved by the local ethics committee under the number EK-Nr. 29–585 ex 16/17 (ethics committee of the Medical University Graz). We included some data from our “Fatty tissue distribution measurement using magnetic resonance imaging study” for this manuscript [24]. Additional blood tests and laboratory measurements were performed in the same cohort. Written informed consent was obtained from all participants before enrolment. In this cross-sectional study, we recruited 180 healthy volunteers (90 males, 90 females, age: 44.71 ± 10.96 (mean ± SD years)). Volunteers with known lipid disorders, metabolic disorders such as diabetes, muscle diseases, neurological disorders or chronic diseases as well as a regular intake of cholesterol-reducing medication or hormones were excluded prior to the study. We obtained anthropometric measurements in every subject including height, waist and hip circumferences using an inelastic measuring tape. All volunteers were weighed using the same scale to avoid bias. The parameters waist/hip ratio and waist/height ratio were calculated using MS Office Excel Version 2021 (Office 365, Microsoft Cooperation, One Microsoft Way, Redmond, 98052-6399, Washington, DC, USA).

All enrolled study participants answered a self-developed questionnaire on pre-existing conditions and lifestyle. They indicated the frequency, endurance and nature of their sporting activities as well as their dietary style. The weekly training frequency and durance for both resistance and endurance training were collected.

### 2.2. Laboratory Analyses: Lipid Parameters and Total Antioxidative Capacity

Venous blood samples were collected from every subject prior to MRI measurements. The blood samples were collected after an overnight fasting period of 12 h. The serum lipid analyses contained total blood cholesterol (mg/dL), HDL (mg/dL), LDL (mg/dL) and serum triglycerides(mg/dL). Total cholesterol/HDL quotient was calculated using MS Office Excel 2016^®^ (Microsoft Cooperation, Washington, DC, USA). All blood samples were analyzed in the same laboratory with a standardised protocol using frozen serum (−80 °C).

The total antioxidative capacity (TAC) of serum samples was determined by assessing the sample’s ability to inhibit 2,2′-azobis(2-amidinopropane) dihydrochloride (AAPH)-induced oxidation of dihydrorhodamine (DHR) as described [25]. Samples were pre-diluted 1:10 in phosphate-buffered saline. AAPH, as well as the fluorescent DHR, were added to the assay buffer (20 mM of HEPES, 150 mM of NaCl, 10 g/L of Chelex-100, 1 mM of AAPH, 7.5 μM DHR, pH of 7.4) in the absence or presence of the pre-diluted serum (final dilution 1:180) samples. Fluorescence intensity was measured, and readings (excitation wavelength, 485 nm; emission wavelength, 538 nm) were performed every 5 min for 60 min. Finally, the AOC per sample was calculated as percentage inhibition in fluorescence per minute due to oxidation of DHR after addition of the sample compared to blank assay buffer.

### 2.3. Laboratory Measurements: LTL

Genomic DNA was prepared from stored EDTA whole blood using a MagNA Pure instrument (Roche, Vienna, Austria). RTL was measured by a qPCR assay developed by Cawthon [26]. The assay quantified the ratio of average telomere length to a single-gene copy (RPLP0, previously denoted as “36B4”). All qPCR analyses were performed in triplicate in 96-well plates on a CFX96 Real-Time PCR Detection System (Bio-Rad Laboratories, Vienna, Austria). Total reaction volume was 25 µL. Thermal cycling profile was 10 min at 95 °C followed by 40 cycles of 15 sec at 95, 30 sec at 54 °C and 30 sec at 72 °C with fluorescence data collection. Each run included a standard curve made by dilutions of available HeLa-DNA (New England Biolabs, Frankfurt, Germany) to determine the quantity of targeted templates in each sample relative to the HeLa-DNA. RTL was calculated as the ratio of telomere quantity to single-copy gene quantity. PCR efficiencies were 106% for telomere amplification and 94% for single-copy gene amplification. Mean intra-assay coefficient of variability (CV) was 8.7%, and plate-to-plate CV was 12.7%.

### 2.4. Statistics

Statistics were performed using RStudio–R version 4.0.2 (Integrated Development for R. RStudio, PBC, Boston, MA, USA). Statistical comparison of parametric continuous subject characteristics was accomplished using the 2-sided Student’s *t*-test with Pearson’s linear correlation coefficients over the whole study population and additionally, separated in group according to sex. Distribution was tested for normality using the Shapiro–Wilk test. A *p*-value < 0.05 was considered to be statistically significant. Furthermore, we performed multiple linear regression analysis with TL as a constant, and included three different models, first adjusted for age, endurance training, resistance training (Table 3a) and additionally adjusted for anthropometric measurements such as BMI, waist/height, and waist/hip as independent variables (Table 3b). Finally, they were adjusted for serum lipid profile (Table 3c).

## 3. Results

### 3.1. Subjects

A total of 180 healthy subjects (90 male, age 40.77 ± 11.62 and 90 female, age 44.71 ± 10.96 (mean ± SD years)) were included in this study. Table 1 shows all anthropometric measurements, body mass index (BMI), height, weight, waist and hip circumferences, LTL, TAC and additional laboratory parameters (lipid profile and adiponectin) of the entire study population. The parameters are indicated for both groups of sex.

There was no statistically significant difference between the two groups of sex concerning the LTL (females: 0.69 ± 0.31 versus males 0.70 ± 0.28/*p* = *0*.75). Additionally, the TAC showed comparable values between men (60.34 ± 8.02) and women (61.66 ± 7.08/*p* = 0.24), whereas average total blood cholesterol was lower in the male group, in contrary to the higher HDL-C in females (75.76 ± 18.04 mg/dL versus 60.20 ± 13.86 mg/dL/*p* = 0.01). The values are reported in mean ± SD. The weekly endurance training time between women and men showed no statistically significant difference. However, on average, women spent less time on strength training than men (Table 1).

### 3.2. Pearson Correlations

**Figure 1:**
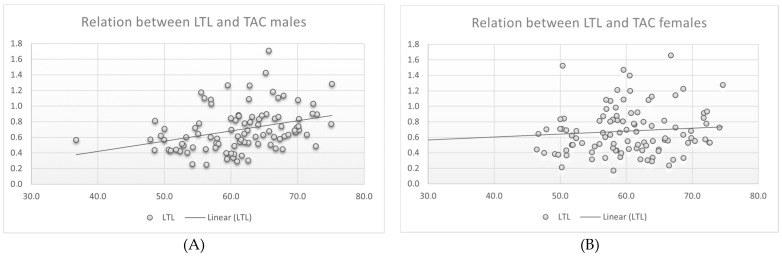
(**A**) Scatterplot of LTL versus TAC data in males, (**B**) Scatterplot of LTL versus TAC data in females.

The main result of our study was a positive significant relation of the LTL and the serum TAC in the male group (r = 0.34/*p* = 0.001). There was no statistically significant correlation between age and LTL in either group. Furthermore, we could not find a significant relationship between anthropometric measurements and the weekly training times of each endurance or strength. (Table 2). Interestingly, we could not find a correlation between LTL and TAC in the female group. Table 2 shows the Pearson correlation between anthropometric measurements, lipid parameters, LTL and TAC in a separate evaluation of both sexes. A scatterplot of the LTL versus TAC data in the male and female group is demonstrated in Figure 1A,B. (Figure 1).

### 3.3. Multiple Regression Analysis

A multiple linear regression analysis positively confirmed our observation. We calculated three different models in the male group first, adjusted for age and the weekly training time for endurance and weight training (Table 3a). Additionally, we adjusted the relation for anthropometric measurements (Table 3b) and serum lipid parameters (Table 3c), in the female group (Table 4a–c).

**Figure 2:**
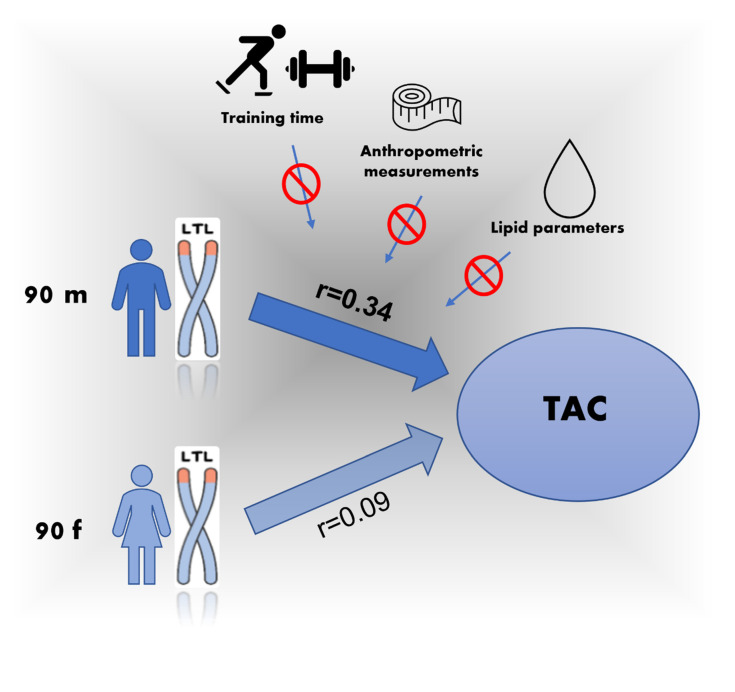
A graphical overview of the correlation between TAC and LTL in males and females.

Figure 2 gives a graphical overview of the relationship between males and females.

## 4. Discussion

The main observation in our study was a significant correlation of LTL and serum TAC in the male group (r = 0.34; *p* = 0.001). This relationship could be substantiated in a multivariate linear regression analysis including age and the weekly training time for endurance and weight training. Interestingly, we did not observe the same relationship in the female group, and no significant effect of serum lipid profile or anthropometric measurement could be found on this correlation.

To explain our observation, one can consider different hypotheses. Firstly, previous studies in humans showed that females have generally longer telomeres than men, and this effect increases with age [27]. We could not find any difference between the LTL between females and males in our study population. The reason for this might be that our cohort consisted of a comparatively young and athletic population, and sex-specific differences may only become apparent with increasing age. A prior study on sheep by Watson et al. indicated that females and males start out with similar telomere lengths in early life, but sex differences emerge in adult sheep and increase in magnitude with age [28].

Secondly, one might consider that telomere length and oxidative stress could vary between men and women due to differences in metabolic health. Our results demonstrated no sex difference in terms of TAC, despite differences in anthropometric measures such as BMI, waist/height and waist/hip ratio.

This is also contrary to previous investigations, as higher levels of oxidized LDL have been confirmed in males. Additionally, higher markers of central adiposity (waist/height ratios) were related to a lower TAC [29]. However, markers of central adiposity are normally higher in males because males store more adipose tissue in the abdominal region [30]. Other studies found an association between metabolically healthy and unhealthy obesity phenotypes with parameters of oxidative stress such as TAC and total oxidation status (TOS) as well as relative TL in healthy young adult men [31].

We found significantly higher serum HDL-C levels in the female group compared to the male group. This is expected, as prior studies described lower LDL-C levels and higher HDL-C levels in women during the reproductive period compared to an age-matched male group [32,33]. Previous studies have shown that female patients with polycystic ovary syndrome (PCOS), for example, show increased indicators of oxidative stress and that this is associated with lowered HDL-C and increased LDL-C levels. For this reason, it could be suggested that the correlation of serum TAC with LTL might also be influenced by serum lipids [34]. 

However, our study cohort was not designed to include unhealthy obese subjects, where these effects are common.

Nevertheless, sex differences concerning diseases involving oxidative stress in males are known, e.g., ischemic heart disease, as well as sex differences in stress response of cells and tissues.

However, there may be another reason why female cells in general seem to be more resistant to oxidative stress. The underlying mechanism might be a beneficial effect of oestrogen as well as a superior mitochondrial function. Female cells were found to show an increased expression of stress response genes compared to male cells [35].

Due to the increased sensitivity of male cells to oxidative stress and of telomeres to hydroxyl radicals [11], their telomeres may shorten faster with increasing age, which could be an explanation for the sex-related difference in TL. Consequently, men in particular might benefit from a high nutritive antioxidant capacity and from a reduction in cardiovascular risk factors in order to positively influence their telomere length. The strongest evidence for modifying the antioxidant capacity of serum is found in the Mediterranean diet. The Mediterranean diet is rich in both vitamins and polyphenols, which are mainly, but not exclusively, contained in fruits, vegetables, whole grains, nuts, extra virgin olive oil and red wine [36]. In line with that assumption, higher concentration of lutein, zeaxanthin and vitamin C in plasma were found to be associated with longer LTL in normal elderly persons and suggest a protective role of these antioxidants in telomere maintenance [37]. Having observed a robust correlation between antioxidant capacity and LTL in healthy young to middle-aged men, further studies should be made with older and obese subjects.

Furthermore, we could not find a correlation of TL with BMI and W/height ratio in our study cohort. One reason might be the limitation to LTL measurement, which is generally not optimized as a surrogate marker for telomere length in other tissues [38,39]. Moreover, LTL represents the average telomere length across all leucocytes, which are composed of different cell types such as granulocytes and lymphocytes. These subpopulations have different average telomere lengths [39]. Furthermore, our cohort consisted of healthy subjects who were on average younger adults leading a more active lifestyle. We assume the difference would only become apparent when a cohort such as this is compared with very obese or diseased subjects. Moreover, TL in general shows high inter-individual variety, and we were not able to measure LTL changes over time in this study.

Additionally, there was no relationship between telomere length and exercise time for either endurance or strength training, which we would have expected after some studies showed telomere lengthening in individuals through measures such as an exercise program [18,23]. A recent study indicated an upregulation in telomerase activity, especially in blood mononuclear cells, due to endurance training and interval training after 6 month of lifestyle intervention but not with resistance training (RT). Further, lymphocyte, granulocyte and leucocyte TL increased in the endurance-training groups but not in the RT group of that study [40].

Besides the restricted availability of only having measured LTL in our study, there were some other limitations. The sex hormone status was not recorded, which could influence LTL, especially in postmenopausal women [41]. Another limitation is that we were not able to measure telomerase activity. We have to note that the results of our present cross-sectional study are observational and therefore preliminary in some aspects. They should be confirmed in longitudinal cohort studies with a larger number of subjects. The sporting activities were collected using a defined questionnaire. In order to perform more accurate studies, future investigations should be performed with performance diagnostics.

To investigate the underlying mechanisms of these relationships and differences, it would be particularly useful to conduct experiments targeting oxidative stress and sex differences in the telomeric structure and sequence in the future. Obviously, a high density of guanine, such as in the telomeric regions, are known to give rise to higher order structure G-quadruplexes [42,43]. Oxidation-potential guanine nucleobases also represent hotspots for oxidative lesions [43,44].

## 5. Conclusions

To the best of our knowledge, this is the first cross-sectional study which indicates an association of total serum TAC and LTL in a healthy young to middle-aged male cohort.

One reason for the observed sex difference could be a variation in the telomeric structure between males and females, which should be investigated in the future.

Another factor for why this result could not be observed in the female group may lie in the sex difference of cellular response to oxidative stress. Our findings suggest that men in particular could benefit from a high antioxidant capacity to increase their likelihood of healthy ageing. The shortening of LTL is associated with increasing age and with age-related diseases such as cardiovascular disease, diabetes mellitus and cancer. It could be suggested that an increase in antioxidant capacity has the potential to prevent or influence the course of numerous common diseases that are among the major causes of mortality and morbidity in aging societies.

## Figures and Tables

**Table 1:** **Descriptive statistic of the study population:** Relative leucocyte telomere lengths and anthropometric measurements are shown along with lipid parameters and duration of sport. Sex differences determined by Welch’s *t*-test are marked with *p*-values. (Values are reported as mean ± SD).

Anthropometric Measurements	Females (*n* = 90)	Males (*n* = 90)	*p* Value
Age	44.71 ± 10.96	40.77 ± 11.62	0.020 *
Weight (kg)	63.38 ± 9.13	80.42 ± 9.97	0.000 ***
Height (cm)	167.11 ± 5.65	180.82 ± 5.93	0.000 ***
Waist/height ratio	0.46 ± 0.05	0.49 ± 0.05	0.000 ***
Waist/hip ratio	0.78 ± 0.06	0.86 ± 0.07	0.000 ***
BMI	22.7 ± 3.17	24.58 ± 2.63	0.000 ***
**Telomeres**			
Relative telomere length	0.69 ± 0.31	0.70 ± 0.28	0.747
**Total antioxidative capacity**			
Mean inhibition of oxidation	60.34 ± 8.02	61.66 ± 7.08	0.242
**Lipid parameters**			
Total cholesterol (mg/dL)	198.06 ± 33.16	189.60 ± 33.89	0.092 *
LDL cholesterol (mg/dL)	101.64 ± 31.40	104.74 ± 32.29	0.516
HDL cholesterol (mg/dL)	75.76 ± 18.04	60.20 ± 13.86	0.000 ***
Triglycerides (mg/dL)	103.66 ± 93.44	123.57 ± 76.41	0.119
Total cholesterol/HDL-C ratio	2.74 ± 0.98	3.35 ± 1.09	0.000 ***
Adiponectin (µg/mL)	13.60 ± 5.36	18 ± 3.71	0.000 ***
**Sports**			
Endurance training (min/week)	183.83 ± 383.42	231.83 ± 317.81	0.363
Strength training (min/week)	71.48 ± 84.14	101.47 ± 115.58	0.048 *

Body mass index (BMI), low-density lipoprotein (LDL); high-density lipoprotein (HDL), * p < 0.05, *** p < 0.001; n = 180.

**Table 2:** **Pearson correlation of relative leucocyte telomere length with body measurements, lipid profile, total antioxidative capacity and type of sport**, *Pearson correlation coefficients (r-values) and associated p-values are shown*.

Variables	Sex	r-Value	*p*-Value
Age	Female	−0.02	0.864
	Male	0.05	0.624
Total antioxidative capacity	Female	0.09	0.420
	**Male**	**0.34**	**0.001** **
Weight (kg)	Female	0.12	0.268
	Male	0.16	0.130
Height (cm)	Female	0.06	0.591
	Male	0.06	0.578
Waist/height ratio	Female	0.13	0.220
	Male	0.03	0.747
Waist/hip ratio	Female	0.07	0.483
	Male	−0.05	0.641
BMI	Female	0.11	0.305
	Male	0.14	0.204
Total cholesterol (mg/dL)	Female	−0.03	0.775
	Male	−0.11	0.294
LDL-C (mg/dL)	Female	0.06	0.558
	Male	−0.08	0.443
HDL-C (mg/dL)	Female	−0.14	0.194
	Male	0.01	0.926
Triglycerides (mg/dL)	Female	−0.04	0.683
	Male	−0.08	0.432
Total cholesterol/HDL-C ratio	Female	0.16	0.146
	Male	−0.07	0.510
Adiponectin (µg/mL)	Female	−0.19	0.067
	Male	0.16	0.135
Endurance training (min)	Female	−0.12	0.268
	Male	0.03	0.754
Strength training (min)	Female	−0.09	0.398
	Male	0.01	0.925

Body mass index (BMI), low-density lipoprotein (LDL); high-density lipoprotein (HDL), ** *p* < 0.01; *n* = 180.

**Table 3:** This table indicates a multiple linear regression analyses including three different models in the male group, first adjusted for age and sport (3a), additionally adjusted for anthropometric measurements (3b) and serum lipid parameters (3c).

**a. Effect of age and sport on the correlation between total antioxidative capacity and relative telomere length in men**						
*Multiple linear regression coefficients (t-values) and associated p-values are shown. Constant: total antioxidative capacity*						
***Model: **y** = *** $\beta_{0}+\beta_{1}x_{1}+\beta_{2}x_{2}$						
					**adj. R2**	**F-Stat (*p*-Value)**
**Predictor**	**Estimate**	**Std. Error**	**t Value**	**Pr (>|t|)**	**0.08**	3.06 (0.02 *)
Constant	0	0.1	0	1		
Relative telomere length	**0.34**	**0.1**	**3.34**	0.001 **		
Age	−0.02	0.11	−0.16	0.87		
Endurance training	0.06	0.1	0.6	0.55		
Strength training	0.07	0.11	0.71	0.48		
**b. Effect of anthropometric measurements on the correlation between the total antioxidatve capacity and relative telomere length in men**						
*Multiple linear regression coefficients (t-values) and associated p-values are shown. Constant: total antioxidative capacity*						
***Model: **y** = *** $\beta_{0}+\beta_{1}x_{1}+\beta_{2}x_{2}$						
					**adj. R2**	**F-Stat**
						**(*p*-Value)**
**Predictor**	**Estimate**	**Std. Error**	**t Value**	**Pr (>|t|)**	**0.17**	2.79 (0.016 *)
Constant	−0.02	0.11	−0.16	0.88		
Relative telomere length	0.09	0.11	0.79	0.43		
Height (cm)	−0.4	1.38	−0.29	0.77		
Waist/height ratio	0.2	0.62	0.33	0.75		
Waist/hip ratio	0.09	0.33	0.28	0.78		
BMI	0.03	0.17	0.18	0.86		
**c** **. Effect of lipid profile on the correlation between total antioxidatve capacity and relative telomere length in men**						
*Multiple linear regression coefficients (t-values) and associated p-values are shown. Constant: total antioxidative capacity*						
***Model: **y** = *** $\beta_{0}+\beta_{1}x_{1}+\beta_{2}x_{2}$						
					**adj. R2**	**F-Stat**
						**(*p*-Value)**
**Predictor**	**Estimate**	**Std. Error**	**t Value**	**Pr (>|t|)**	**0.22**	3.27 (0.004 **)
Constant	0	0.1	−0.01	0.99		
Relative telomere length	**0.36**	**0.1**	**3.6**	0.0005 ***		
Adiponectin (µg/mL)	−0.15	0.1	−1.5	0.14		
Total cholesterol (mg/dL)	−8.28	4.55	−1.82	0.07		
LDL (mg/dL)	7.7	4.32	1.78	0.08		
HDL (mg/dL)	3.63	1.88	1.93	0.06		
Triglycerides (mg/dL)	3.76	2.05	1.84	0.07		
Cholesterol/HDL ratio	0.15	0.37	0.4	0.69		

* *p* < 0.05, ** *p* < 0.01, *** *p* < 0.001; *n* = 90.

**Table 4:** This table indicates a multiple linear regression analyses. We calculated three different models in the female group, first adjusted for age and sport (4a), additionally adjusted for anthropometric measurements (4b) and serum lipid parameters (4c).

**a.: Effect of age and sport on the correlation between total antioxidatve capacity and relative telomere length in women**						
*Multiple linear regression coefficients (t-values) and associated p-values are shown. Constant: total antioxidative capacity*						
***Model: **y** = *** $\beta_{0}+\beta_{1}x_{1}+\beta_{2}x_{2}$						
					**adj. R2**	**F-Stat (*p*-Value)**
**Predictor**	**Estimate**	**Std. Error**	**t Value**	**Pr (>|t|)**	**0.034**	**0.55(0.696)**
Constant	0.01	0.11	0.012	0.906		
Relative telomere length	0.09	0.11	0.81	0.421		
Age	−0.12	0.11	−1.13	0.262		
Endurance training	0.03	0.11	0.26	0.795		
Strength training	0.03	0.12	0.256	0.8		
**b.: Effect of anthropometric measurements on the correlation between total antioxidative capacity and relative telomere length in women**						
*Multiple linear regression coefficients (t-values) and associated p-values are shown.* *Constant: total antioxidative capacity*						
***Model: **y** = *** $\beta_{0}+\beta_{1}x_{1}+\beta_{2}x_{2}$						
					adj. R2	**F-Stat**
						**(*p*-Value)**
**Predictor**	**Estimate**	**Std. Error**	t Value	Pr (>|t|)	0.13	**2.79 (0.821)**
Constant	0	0.11	0	1		
Relative telomere length	0.07	0.11	0.6	0.553		
Height (cm)	0.03	0.6	0.05	960		
Waist/height ratio	0.04	0.33	0.13	0.894		
Waist/hip ratio	0.07	0.17	0.41	0.683		
BMI	0.1	1.34	0.08	0.938		
**4c.: Effect of lipid profile on the correlation between total antioxidative capacity and relative telomere length in women**						
*Multiple linear regression coefficients (t-values) and associated p-values are shown. Constant: total antioxidative capacity*						
***Model: **y** = *** $\beta_{0}+\beta_{1}x_{1}+\beta_{2}x_{2}$						
					**adj. R2**	**F-Stat (*p*-Value)**
**Predictor**	**Estimate**	**Std. Error**	**t Value**	**Pr (>|t|)**	**0.1**	**1.26 (0.299)**
Constant	0.03	0.11	0.24	0.811		
Relative telomere length	0.07	0.10	0.61	0.546		
Adiponectin (µg/mL)	−0.22	0.11	−1.95	0.055		
Total cholesterol (mg/dL)	−2.59	2.12	−1.22	0.226		
LDL (mg/dL)	2.10	1.86	1.13	0.26		
HDL (mg/dL)	1.46	1.27	1.15	0.254		
Triglycerides (mg/dL)	1.59	1.24	1.28	0.205		
Cholesterol/HDL ratio	0.24	0.43	0.57	0.57		

## Data Availability

The data presented in this study are openly available in Fig Share at https://figshare.com/articles/dataset/Dataset_Telomere_Tac_xlsx/16817455/1 (accessed on 2 November 2021) [45].
